# Neuroprotection and Enhanced Hemoglobin Excretion by Intrathecal Clazosentan in Mice With Subarachnoid Hemorrhage

**DOI:** 10.1161/JAHA.126.049695

**Published:** 2026-08-07

**Authors:** Fumihiro Kawakita, Koichi Hakozaki, Kazuaki Aoki, Hidenori Suzuki

**Affiliations:** ^1^ Department of Neurosurgery Mie University Graduate School of Medicine Tsu Japan

**Keywords:** clazosentan, early brain injury, hemoglobin, subarachnoid hemorrhage, Intracranial Hemorrhage

## Abstract

**Background:**

Intravenous administration of clazosentan, a selective endothelin receptor subtype A antagonist, has been used as an anti‐vasospastic agent. This study aimed to investigate whether intrathecal administration of clazosentan prevents neuroinflammation and affects lymphatic clearance of hemoglobin in a mouse model of acute subarachnoid hemorrhage (SAH).

**Methods:**

Male C57BL/6 mice (n=109) underwent prechiasmatic cisternal injection models of SAH or sham models. From 30 minutes post‐modeling, mice randomly received a continuous intracerebroventricular infusion of saline or clazosentan (1, 0.2, and 0.04 μg in 192 μL of saline; defined as 50%, 10%, and 2% of the clinically achievable blood concentration, respectively) at a rate of 8 μL/h for 24 hours. The intervention effects were assessed by neurobehavioral testing, SAH grading, and immunohistochemical staining at 24 and 48 hours, and Western blotting at 24 hours post‐modeling.

**Results:**

Clazosentan treatment did not influence SAH grading but showed a dose‐dependent trend toward improved survival in SAH mice. A comparison of saline treatment and 50% clazosentan treatment revealed that clazosentan significantly suppressed post‐SAH neurological deterioration, hemoglobin infiltration into the brain, and induction of ionized calcium‐binding adapter molecule 1 and tenascin‐C in the brain, and significantly upregulated vascular endothelial growth factor receptor‐3 in the brain without affecting vascular endothelial growth factor‐C levels. When comparing the effects of different clazosentan doses, an inhibitory effect on neuroinflammation was observed up to 10% clazosentan. At 48 hours post‐modeling, clazosentan administration dose‐dependently inhibited neurological deterioration, hemoglobin infiltration into the brain and activation of perivascular macrophages and microglia, and increased hemoglobin deposition in the deep cervical lymph nodes of SAH mice without intracranial lymphatic vessel proliferation.

**Conclusions:**

This study first showed that intrathecal administration of clazosentan can suppress early brain injury in terms of neuroinflammation at cerebrospinal fluid concentrations one‐tenth of the clinically achievable blood concentrations, associated with enhanced lymphatic drainage of hemoglobin.

## Graphical abstract

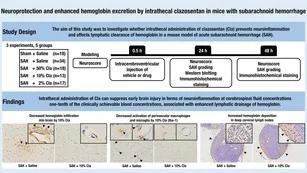

**Nonstandard Abbreviations and Acronyms**
BW: body weight
Cla: clazosentan
CSF: cerebrospinal fluid
EBI: early brain injury
ETR_A_
: endothelin receptor subtype A
Hb: hemoglobin
Iba‐1: ionized calcium‐binding adapter molecule 1
LN: lymph node
SAH: subarachnoid hemorrhage
TNC: tenascin‐C
VEGFR: vascular endothelial growth factor receptor

**Research Perspective**

**What Is New?**

- This study replicated clinically observed cerebrospinal fluid concentrations of clazosentan, a selective endothelin receptor subtype A antagonist, by intrathecal administration of the drug, and first demonstrated that intrathecal clazosentan suppressed early brain injury in a mouse model of acute subarachnoid hemorrhage by inhibiting neuroinflammation.
- The neuroprotective effect of intrathecal administration of clazosentan in a mouse subarachnoid hemorrhage model was accompanied by enhanced lymphatic drainage of hemoglobin.

**What Question Should Be Addressed Next?**

- Is intrathecal administration of clazosentan more effective than intravenous administration in removing hemoglobin from cerebrospinal fluid and exerting neuroprotective effects?

Subarachnoid hemorrhage (SAH) caused by aneurysmal rupture remains poor in terms of outcome.^1^ Basic research has focused on early brain injury (EBI) to elucidate its pathophysiology and develop novel treatments. EBI occurs within 72 hours after SAH onset and is considered a major factor determining SAH outcomes.^2^ One of the key triggers of EBI is free hemoglobin (Hb).^3^ Hb is released after lysis of erythrocytes in the cerebrospinal fluid (CSF). Hb invades the perivascular space within the brain parenchyma and brain tissue, causing neuroinflammation and upregulating the expression of a matricellular protein tenascin‐C (TNC).^3^, ^4^ TNC has been repeatedly reported to cause not only cerebral vasospasm but also EBI.^2^, ^4^ Our recent study showed that a Hb scavenger protein haptoglobin prevented EBI and cerebral vasospasm in SAH mice by inhibiting Hb infiltration into the brain parenchyma and increasing Hb drainage into the lymphatic system.^5^ However, its effects on TNC expression levels and lymphatic vessel proliferation have not been investigated. Lymphangiogenesis after stroke is an important topic that has recently attracted attention, but it remains unclear whether lymphangiogenesis is neuroprotective or promotes neuronal injury.^6^, ^7^

Recently, intravenous infusion of clazosentan (Cla), a selective endothelin receptor subtype A (ETR_A_) antagonist, has been used as an anti‐vasospastic agent in clinical settings in Japan. The results of randomized controlled trials of Cla for SAH differed between Japan and the West.^8^, ^9^ The exact reasons for this are unclear, but it is thought that intravenous infusion of Cla may not have been effective in preventing cerebral microcirculatory disorders, or that systemic side effects such as pulmonary edema and hypotension may have offset the beneficial effects of Cla.^10^, ^11^ Because Cla has a relatively large molecular weight and does not readily cross the blood–brain barrier under physiological conditions,^12^ intrathecal infusion of Cla may not only be less likely to cause systemic complications but may also have a greater effect on post‐SAH pathophysiology than intravenous administration. Pericytes possess ETR_A_ and are known to be involved in small vessel constriction and spreading depolarization, which are important components of EBI and vasospasm‐unrelated delayed cerebral ischemia.^3^, ^13^ Perivascular macrophages also possess ETR_A_ and are involved in the function of the glymphatic system.^14^, ^15^ Therefore, the authors hypothesized that intrathecal infusion of Cla may suppress EBI after SAH. The aim of this study was to investigate whether intrathecal infusion of Cla could prevent EBI from the perspective of neuroinflammation and affect TNC expression levels, lymphatic clearance of Hb, and lymphatic proliferation in SAH mice.

## METHODS

The authors declare that all supporting data are available within the article and its online supplemental files (see Data S1).

### Animals

All procedures were approved by our institution's Animal Ethics Review Committee and were carried out in accordance with the institution's guidelines and the ARRIVE (Animals in Research: Reporting In Vivo Experiments) guidelines. Male C57BL/6 mice (10–12 weeks old, weighting 25–30 g; SLC, Hamamatsu, Japan) were housed in a temperature‐ and humidity‐controlled room with a 12‐hour light and dark cycle and provided with free access to food and water. Because this study aimed to clarify whether intrathecal microadministration of Cla affects cerebral pathophysiology after SAH, we used only male animals. The sample size of this study (n=6 or 12 per group) was determined based on a large effect size (≥0.80) expected from preliminary data and previous studies using similar models.^5^, ^15^ After modeling, animals were immediately randomized by lottery until the final group size reached the planned number of experiments. Data collection and analysis were performed in a blinded manner.

### Drug

In Japan, Cla (Nxera Pharma, Tokyo, Japan) is administered intravenously at a rate of 10 mg/h,^16^ resulting in a blood concentration of approximately 0.5 μg/mL in a 60 kg person (blood volume approximately 4374 mL). To achieve this concentration in the CSF of a mouse, a 25 g mouse (40 μL CSF volume) would require a calculated Cla dose of 0.0914 μg/h. To achieve a 24‐hour continuous infusion at this dose, a total Cla dose of 2.194 μg would be dissolved in 192 μL of solvent (physiological saline) and administered at a rate of 8 μL/h. Therefore, a 24‐hour total Cla dose of 2 μg/25 g body weight (BW) (80 μg/kg BW) was defined as 100% Cla. There have been no previous reports on Cla concentrations in CSF in mice. In our preliminary clinical study, CSF concentrations of Cla were less than 50% of the blood concentration (data not shown). In this study, 1, 0.2, and 0.04 μg of Cla was diluted with 192 μL of vehicle (sterile physiological saline) and administered as 50%, 10%, and 2% Cla, respectively.

### SAH Model and Study Protocol

The prechiasmatic cisternal injection model of SAH was produced as previously described.^17^ Each animal was anesthetized with a combination of midazolam (4 mg/kg BW), medetomidine (0.75 mg/kg BW), and butorphanol tartrate (5 mg/kg BW) administered intraperitoneally at 10 mL/kg BW. After the head was fixed in a stereotactic frame, a burr hole was drilled 4.5 mm anterior and 0.5 mm to the right of bregma. A 27‐gauge needle was stereotaxically inserted into the prechiasmatic cistern at a 40° angle from the burr hole, and 100 μL of non‐heparinized autologous blood collected from the tail was manually injected over 15 seconds. For sham surgery, 100 μL of physiological saline was similarly injected into the prechiasmatic cistern.

After modeling, a small subcutaneous pocket was created on the mouse's back by peeling the subcutaneous connective tissue toward the back through a midline skin incision to expose the skull. Another burr hole was then drilled 0.2 mm posterior and 1.0 mm to the right of bregma.^18^ A ventricular cannula was connected to an ALZET mini‐osmotic pump (Model #2001D; Durect Corp, Cupertino, CA) filled with drug solution. The mini‐osmotic pump was inserted into the subcutaneous pocket, and the ventricular cannula was inserted through the burr hole to a depth of 2.5 mm into the right lateral ventricle.^18^ The skin incision was closed with sutures, and the ventricular cannula and mini‐osmotic pump were placed completely subcutaneously. After surgery, mice were awakened from anesthesia, returned to their cages, and allowed to eat and drink ad libitum. Drug solution was continuously and automatically infused intracerebroventricularly via the subcutaneously inserted mini‐osmotic pump (Model #2001D; Durect Corp, Cupertino, CA) at a rate of 8 μL/h for 24 hours after surgery (from 30 minutes to 24.5 hours after SAH modeling), regardless of the animal's BW. Body temperature was maintained at 37 °C, and blood pressure, heart rate, and oxygen saturation were measured noninvasively from the tail throughout the surgery.

All animals were used, and no exclusions were made. This study consisted of three experiments (Figure S1; Table S1). First, to evaluate the neuroprotective effects of Cla, 39 animals were randomly divided into the sham+saline (n=12), SAH+saline (n=15), and SAH+50% Cla (n=12) groups. At 24 hours post‐modeling, neurobehavioral testing and SAH grading were assessed, followed by Western blotting (Hb, TNC, ionized calcium‐binding adapter molecule 1 [Iba‐1], vascular endothelial growth factor [VEGF]‐C, and VEGF receptor [VEGFR]‐3) and immunohistochemical staining (Hb) of the brain.

Next, to examine the dose–response relationship of Cla to SAH toxicity, 28 SAH mice were intracerebroventricularly injected with saline (n=8), 50% Cla (n=6), 10% Cla (n=6), or 2% Cla (n=8), and neurobehavioral testing, SAH grading and Western blotting (Hb, TNC, and Iba‐1) were performed 24 hours after modeling.

Third, to confirm the neuroprotective effect of Cla and examine its relationship to lymphatic drainage, 33 mice were randomly divided into the following groups: sham + saline (n=6), SAH+saline (n=11), SAH+10% Cla (n=7), and SAH+2% Cla (n=9) groups. Neurobehavioral testing was performed 24 and 48 hours after modeling, and SAH grading and immunohistochemical staining of the brain (Hb, Iba‐1, VEGFR‐3, and lymphatic vessel endothelial hyaluronan receptor 1) and deep cervical lymph nodes (LNs; Hb) were evaluated 48 hours after modeling.

### Neurobehavioral Testing

The 6‐item modified Garcia neurological scoring system was used, and neurobehavior was assessed in a blinded manner as previously described.^19^

### SAH Grading

The severity of SAH was assessed in a blinded manner at each euthanization as previously described.^19^ The basal and prepontine cisterns were divided into 6 segments, and the amount of SAH in each segment was scored on a scale of 0 to 3. The sum of the scores for each segment was used to assess the severity of SAH per animal as the SAH grade.

### Western Blotting

The entire right (blood–injection side) cerebral hemisphere was used for Western blotting as previously described.^5^ Equal amounts of protein samples were electrophoresed and transferred to polyvinylidene difluoride membranes, which were blocked with 5% bovine serum albumin for 2 hours at room temperature, and then incubated overnight at 4 °C with rabbit polyclonal anti‐Hb (LS‐B12169, 1:2000; LSBio, Seattle, WA), monoclonal anti‐Iba‐1 (ab178847, 1:1000; Abcam, Cambridge, MA), polyclonal anti‐TNC (1:1000; produced as previously described^20^), polyclonal anti‐VEGF‐C (ab9546, 1:500; Abcam, Cambridge, MA), and polyclonal anti‐VEGFR‐3 (ab27278, 1:1000; Abcam, Cambridge, MA) antibodies. β‐Tubulin (2146, 1:4000; Cell Signaling Technology, Danvers, MA) was blotted onto the same membrane as a loading control. Immunoreactive bands were detected with a chemiluminescence reagent kit (ECL Prime; Amersham Bioscience, Arlington Heights, IL) and quantified by densitometry using Image J software. Each protein level was expressed as a ratio to β‐tubulin levels for normalization.

### Immunohistochemical Staining

Brains and deep cervical LNs fixed in 10% neutral buffered formalin were used for immunohistochemical staining as previously described.^5^ Sections were prepared at a thickness of 4 micrometers. Brains were sectioned coronally 1.0 mm posterior to the bregma. After dewaxing and rehydration, the sections were subjected to antigen retrieval by heating in 1 mmol/L EDTA (pH 8.0) at 80 °C for 20 minutes. After quenching endogenous peroxidase activity by incubation with 1% hydrogen peroxide for 10 minutes, sections were blocked with 10% normal goat serum for 1 hour at room temperature and incubated with rabbit polyclonal anti‐Hb (LS‐B12169, 1:100; LSBio, Seattle, WA), monoclonal anti‐Iba‐1 (ab178847, 1:2000; Abcam, Cambridge, MA), polyclonal anti‐VEGFR‐3 (ab27278, 1:100; Abcam, Cambridge, MA), and polyclonal anti‐lymphatic vessel endothelial hyaluronan receptor 1 (ab14917, 1:100; Abcam, Cambridge, MA) antibodies overnight at 4 °C, followed by incubation with biotinylated goat anti‐rabbit polyclonal immunoglobulin G (1:200; Vector Laboratories, Newark, CA) and then with an avidin‐biotin‐horseradish peroxidase complex (Vectastain Elite ABC Rabbit HPR Kit; Vector Laboratories, Newark, CA). The color reaction was developed with diaminobenzidine/hydrogen peroxide solution, and sections were lightly counterstained with hematoxylin. Hb expression levels were assessed in 4 consecutive 200× magnification images of the right (blood–injection side) temporal cortex (Figure S2) and one 40× magnification image of the LN. Relative amounts of Hb were calculated using Image Pro Plus 6.0 software (Media Cybernetics, Rockville, MD). Hb expression levels in the LNs were calculated in pixels to compensate for differences in LN size.

### Statistical Analysis

Data were analyzed using SPSS version 29 (IBM Corp., Chicago, IL). Mortality rates were compared using the Chi‐square or Fisher exact tests. Neuroscores and SAH grades were expressed as median ±25th–75th percentiles and analyzed using the Mann–Whitney *U* test or Kruskal−Wallis test followed by a Mann–Whitney *U* post‐hoc test with Bonferroni correction, as appropriate. Other values were expressed as mean±SD and compared by 1‐way ANOVA with Student–Newman–Keuls post‐hoc test. The Kaplan–Meier method was used for cumulative survival rates, and the log‐rank test was used to assess statistical significance among SAH study groups. Because of the small sample size, we also performed permutation tests (10 000 iterations) using R version 4.5.3 (R Foundation for Statistical Computing, Vienna, Austria) to validate the *P* values obtained from the above tests. A *P* value of <0.05 was considered significant.

## RESULTS

### Neuroprotective Effects of Cla

In the first experiment, a total of 41 mice underwent SAH or sham modeling, and 2 SAH mice died before randomization (Table S1). No significant differences in physiological parameters were observed among groups (data not shown). None of the sham‐operated mice (n=12) died during the 24‐hour observation period after randomization. The 24‐hour mortality rate of SAH mice after randomization was not significantly different between the SAH+saline (20.0%, 3 of 15) and the SAH+50% Cla (0%, 0 of 12) groups.

At 24 hours after modeling, neuroscores of the SAH+saline group were significantly worse than those of sham‐operated mice, whereas 50% Cla significantly prevented post‐SAH neurological impairment, although the SAH grades were similar between the SAH groups (Figure 1A and 1B). Immunohistochemical staining demonstrated that 50% Cla significantly inhibited post‐SAH increases in Hb infiltration into the perivascular spaces and brain tissue (Figure 1C and 1D).

**Figure 1:**
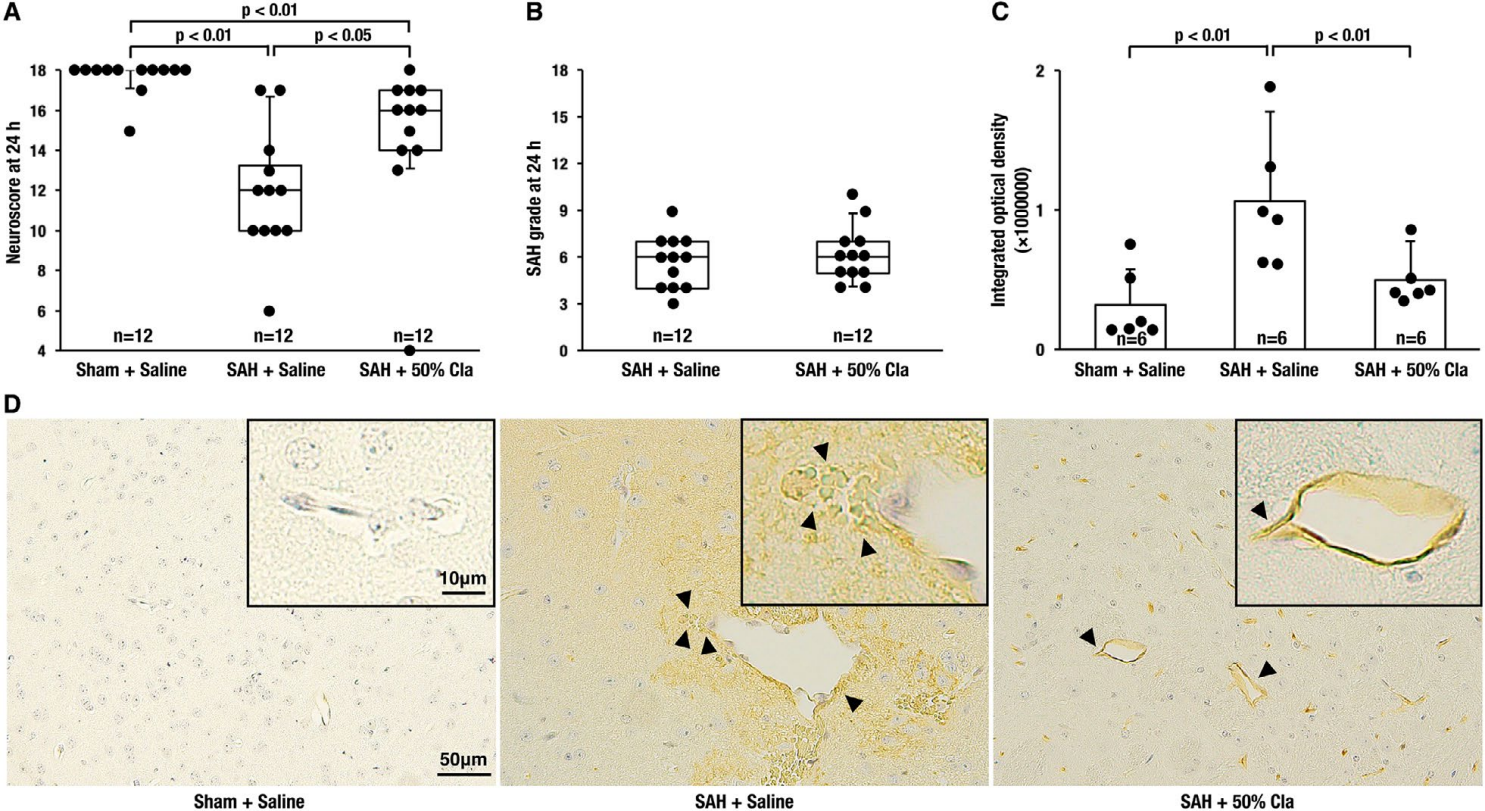
Effects of intrathecal administration of 50% Cla on neuroscores and cerebral hemoglobin infiltration at 24 hours after SAH. **A**, Neuroscore. **B**, Severity of SAH. **C**, Quantitative analysis of hemoglobin infiltration into the right temporal cortex at bregma ‐1 mm. **D**, Representative immunostaining for hemoglobin in the right temporal cortex at bregma ‐1 mm (*inlets* showing magnified small vessels and the perivascular spaces; *arrowheads*, infiltrated hemoglobin in perivascular spaces and brain tissues). Cla treatment has no effects on SAH grades (**B**), but significantly improves neuroscores (**A**) and decreases hemoglobin infiltration (**C**, **D**) compared with the SAH+saline group. Data are expressed as medians ±25th–75th percentiles (**A**, **B**) or means±SD (**C**), and compared using Kruskal−Wallis test (**A**), Mann–Whitney *U* test (**B**) and ANOVA (**C**). ANOVA indicates 1‐way analysis of variance; Cla, clazosentan; and SAH, subarachnoid hemorrhage.

Western blotting of the brain revealed that Hb levels increased after SAH and tended to decrease by 50% Cla treatment: the reason why Hb levels did not decrease significantly with Cla treatment was thought to be because of the influence of hematoma in the subarachnoid space (Figure 2A and 2B). Western blotting also showed that Iba‐1 and TNC were significantly upregulated by SAH and suppressed by 50% Cla (Figure 2C and 2D). In contrast, brain VEGF‐C and VEGFR‐3 levels were not affected by SAH induction, but VEGFR‐3 levels were significantly induced by 50% Cla treatment (Figure 2E and 2F).

**Figure 2:**
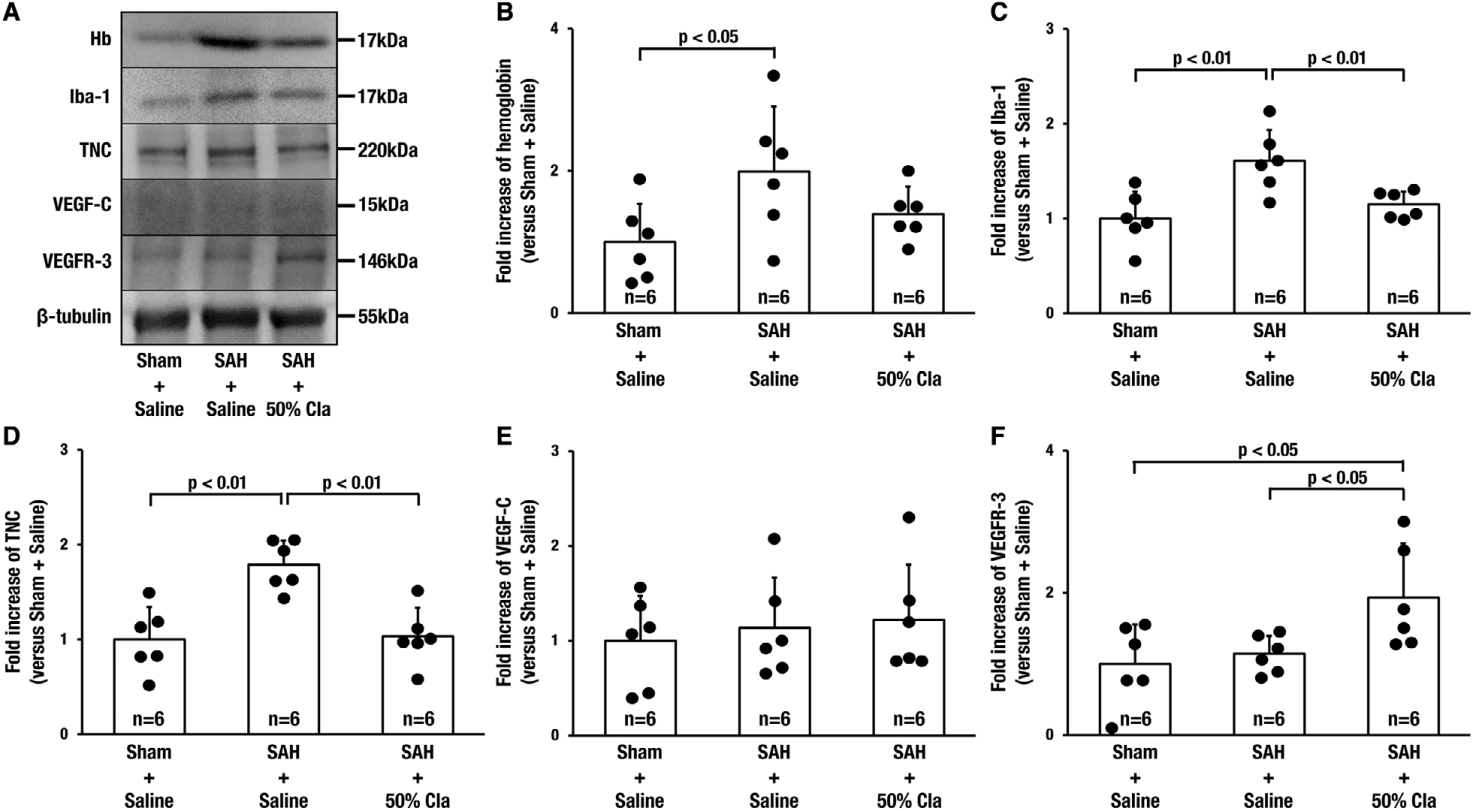
Effects of intrathecal administration of 50% Cla on protein expression in the right cerebral hemisphere at 24 hours after SAH. **A**, Representative Western blots. **B**, Hb. **C**, Iba‐1. **D**, TNC. **E**, VEGF‐C. **F**, VEGFR‐3. Cla prevents post‐SAH increases in Hb infiltration into the brain tissues and upregulation of both Iba‐1 and TNC. Cla also upregulates VEGFR‐3, although SAH has no effects on VEGFR‐3 expression. Expression levels of each protein are expressed as a ratio of ß‐tubulin levels for normalization. Data are expressed as means±SD, and compared using ANOVA. Cla indicates clazosentan; Hb, hemoglobin; Iba‐1, ionized calcium‐binding adapter molecule 1; SAH, subarachnoid hemorrhage; TNC, tenascin‐C; VEGF‐C, vascular endothelial growth factor‐C; and VEGFR‐3, vascular endothelial growth factor receptor‐3.

### Dose–Response Effect of Cla on EBI

The second experiment was conducted using 28 SAH mice to determine the minimum dose of Cla that prevented EBI 24 hours after SAH. Comparison of mortality and physiological parameters revealed no significant differences among groups (Table S1). Neuroscore was significantly better in the 50% Cla‐treated group than in the SAH+saline group and tended to be better in the 10% Cla‐treated group, but the difference was not significant (*P*=0.09), although SAH grades were similar (Figure 3A and 3B). Western blotting of the brain showed no significant differences in Hb levels among groups, but Iba‐1 and TNC levels were significantly suppressed in the 50% Cla‐treated group, and Iba‐1 levels were also significantly suppressed in the 10% Cla‐treated group compared with the SAH+saline group (Figure 3C). Iba‐1 and TNC levels tended to be suppressed by 2% Cla treatment, but the differences from the SAH+saline group did not reach significance. These results suggested that up to 10% Cla was effective against EBI, and therefore the following experiments were conducted using SAH+saline, SAH+2% Cla, and SAH+10% Cla mice.

**Figure 3:**
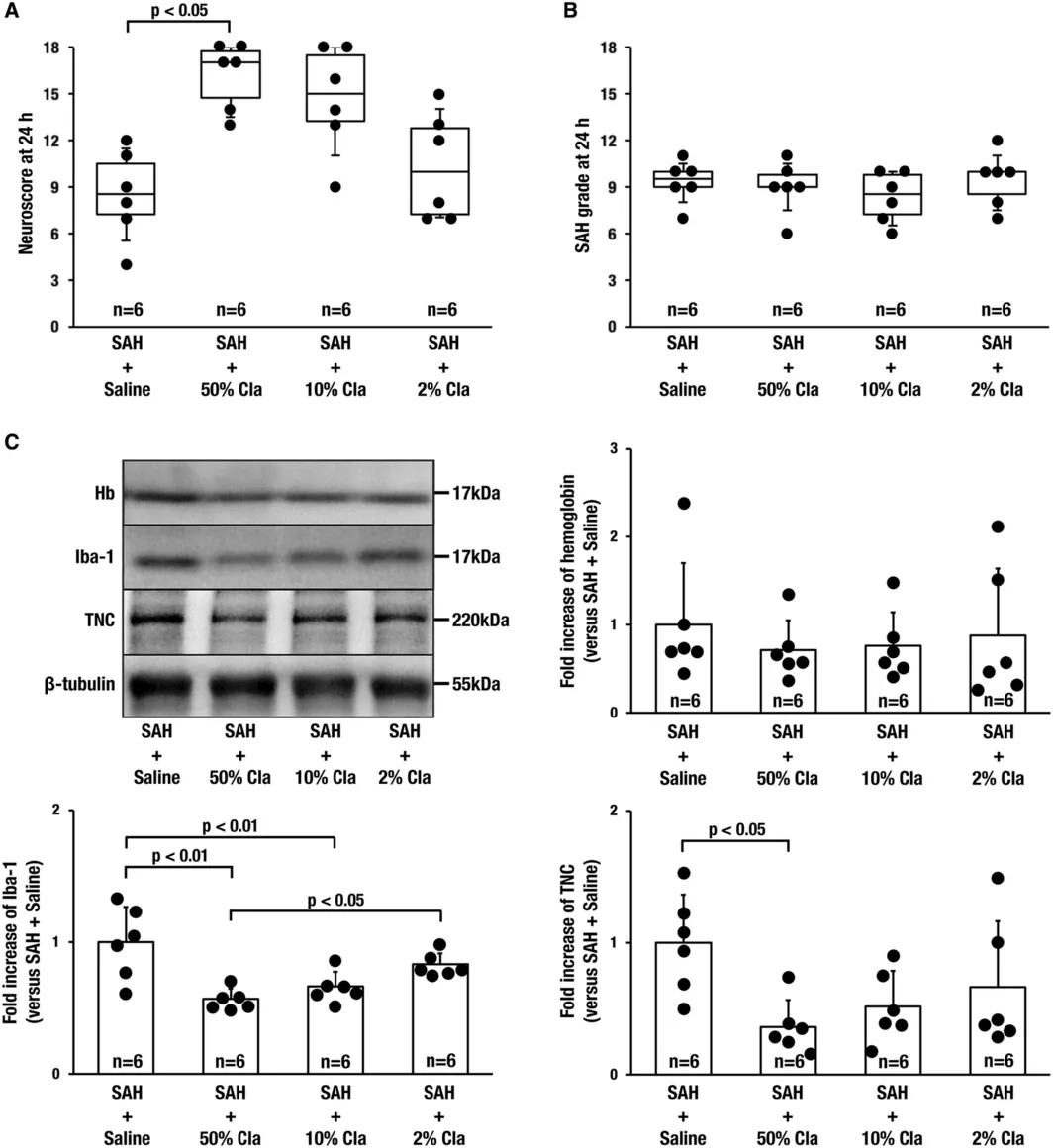
Effects of intrathecal administration of 50% to 2% Cla on brain at 24 hours after SAH. **A**, Neuroscore. **B**, Severity of SAH. **C**, Representative Western blots and quantitative analysis of expression levels of Hb, Iba‐1 and TNC in the right cerebral hemisphere. Cla has no effects on SAH grades and Hb infiltration into the brain tissue, but 50% Cla significantly improves neuroscores and suppresses Iba‐1 and TNC expression compared with the SAH+saline group: 10% Cla also significantly inhibits Iba‐1 expression. Data are expressed as medians ±25th–75th percentiles (**A**, **B**) or means±SD (**C**), and compared using Kruskal−Wallis test (**A**, **B**) and ANOVA (**C**). ANOVA indicates 1‐way analysis of variance; Cla, clazosentan; Hb, hemoglobin; Iba‐1, ionized calcium‐binding adapter molecule 1; SAH, subarachnoid hemorrhage; and TNC, tenascin‐C.

### Confirmation of the Neuroprotective Effect of Cla and its Potential Mechanism

In the third experiment, mortality, physiological parameters, and SAH grades were not significantly different among SAH groups, but administration of 10% Cla significantly prevented neurological deterioration at 24 and 48 hours after SAH (Table S1 and Figure 4A and 4C).

**Figure 4:**
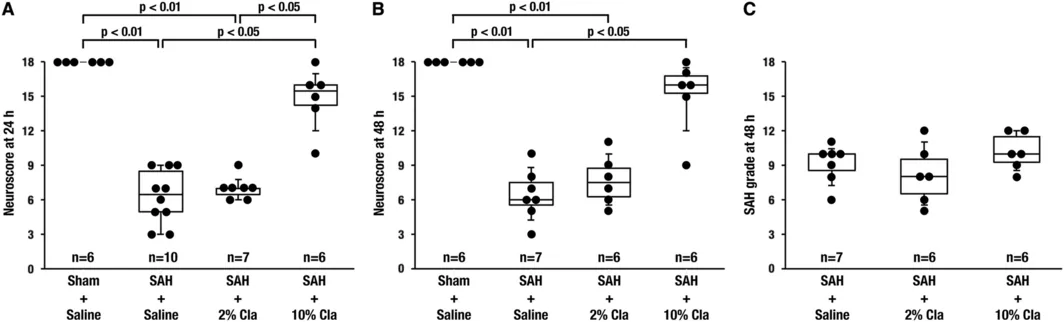
Effects of intrathecal administration of 2% or 10% Cla on neuroscore at 24 hours, neuroscore and the severity of SAH at 48 hours after SAH. **A**, Neuroscore at 24 h. **B**, Neuroscore at 48 h. **C**, Severity of SAH at 48 h. Treatment with 10% Cla significantly improves neuroscores at 24 and 48 h post‐SAH compared with the SAH+saline group (**A**, **B**), although Cla treatment has no effects on SAH grades (**C**). Data are expressed as medians ±25th–75th percentiles, and compared using Kruskal−Wallis test (**A**, **C**). Cla indicates clazosentan; and SAH, subarachnoid hemorrhage.

Immunohistochemical staining 48 hours post‐modeling demonstrated that Hb infiltrated into the perivascular space and brain tissue, and that perivascular macrophages and microglia were activated after SAH. Cla administration dose‐dependently inhibited Hb infiltration and activation of macrophages and microglia (Figure 5A and 5B). Macroscopically, the deep cervical LNs of SAH mice exhibited a red color, whereas those of sham‐operated mice did not. Immunostaining of LNs demonstrated an increase in Hb in lymphatic sinuses after SAH, and Cla administration further increased Hb in LNs in a dose‐dependent manner, suggesting that lymphatic drainage of Hb was promoted (Figure 5C). However, VEGFR‐3 expression was increased by Cla, mainly on the brain surface, whereas lymphatic vessel endothelial hyaluronan receptor 1 expression did not increase even with 10% Cla, and no lymphatic vessel proliferation was observed (Figure 5D and 5E).

**Figure 5:**
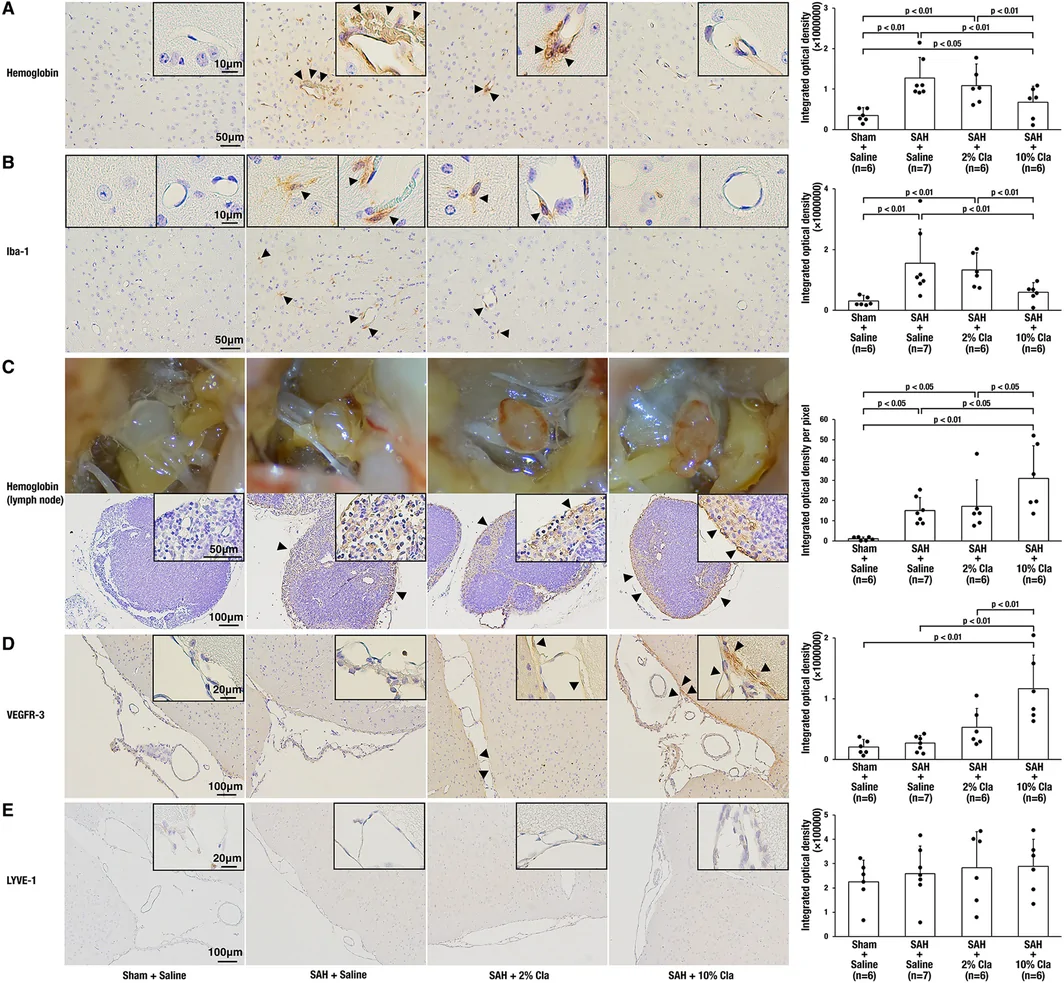
Immunohistochemical staining of the right temporal cortex at bregma ‐1 mm and deep cervical lymph nodes at 48 hours after modeling. **A**, Hemoglobin infiltration into the brain after SAH is significantly suppressed by 10% Cla treatment (*inlets* showing magnified small vessels and the perivascular spaces; *arrowheads*, infiltrated hemoglobin in perivascular spaces and brain tissues). **B**, Post‐SAH increases in Iba‐1 expression is significantly inhibited by 10% Cla treatment (*left inlets*, brain tissues; *right inlets*, perivascular spaces; *arrowheads*, Iba‐1−immunopositive cells [microglia in the *left inlets* and perivascular macrophages in the *right inlets*]). **C**, Post‐SAH increases in hemoglobin deposits in deep cervical lymph nodes are further increased by 10% Cla treatment (*upper*, macroscopic findings; *lower*, hemoglobin immunolabeling; *inlets*, magnified lymphatic sinus; *arrowheads*, hemoglobin infiltration into lymphatic sinuses). **D**, Immunolabeling of VEGFR‐3 is not increased by SAH, but is upregulated mainly on the brain surface by 10% Cla treatment (*inlets*, magnified brain surface; *arrowheads*, VEGFR‐3−immunopositive leptomeningeal cells). **E**, Immunolabeling of LYVE‐1 is neither increased by SAH nor Cla treatment (*inlets*, magnified brain surface). Data are expressed as mean±SD, and compared using ANOVA (**A**−**E**). ANOVA indicates 1‐way analysis of variance; Cla, clazosentan; Iba‐1, ionized calcium‐binding adapter molecule 1; LYVE‐1, lymphatic vessel endothelial hyaluronan receptor 1; SAH, subarachnoid hemorrhage; and VEGFR‐3, vascular endothelial growth factor receptor‐3.

### Survival Benefit from Cla Administration

No adverse effects were observed with Cla administration throughout the study. Combining the results of experiments 1–3, the Kaplan–Meier method showed a dose‐dependent trend toward improved survival in SAH mice treated with Cla (Figure 6).

**Figure 6:**
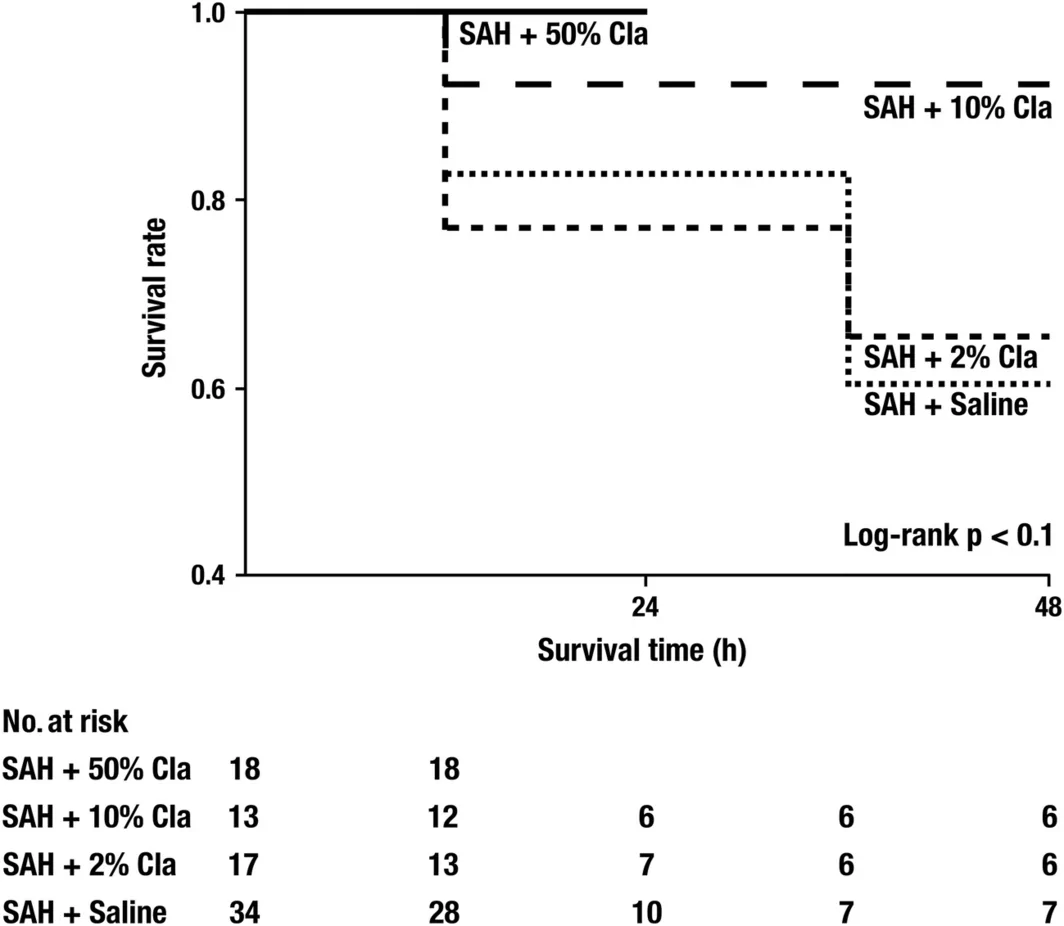
Kaplan–Meier survival estimates demonstrating a dose‐dependent trend toward improved survival in SAH mice treated with Cla. Cla indicates clazosentan; and SAH, subarachnoid hemorrhage.

### Validation Using Permutation Tests

Considering the small sample size, we performed additional permutation tests to validate the robustness of the results. Although the permutation tests did not show statistically significant differences in some items (Figure 1A: neuroscore at 24 hours, SAH+saline versus SAH+50% Cla groups, *P*=0.09; Figure 2: Iba‐1, SAH+saline versus SAH+50% Cla groups, *P*=0.06, and VEGFR‐3, SAH+saline versus SAH+50% Cla groups, *P*=0.07; Figure 3: TNC, SAH+saline versus SAH+50% Cla groups, *P*=0.09; Figure 5: lymph node, SAH+saline versus SAH+10% Cla groups and SAH+2% Cla versus SAH+10% Cla groups, *P*=0.08, respectively), the results supported the overall trends observed in the primary analysis.

## DISCUSSION

The new findings of this study are as follows: (1) post‐SAH intrathecal administration of Cla, a clinically available selective ETR_A_ antagonist, dose‐dependently prevented EBI in terms of neuroscores and neuroinflammation; (2) the neuroprotective effect of Cla was associated with reduced Hb infiltration into the intraparenchymal perivascular space and brain tissue and enhanced lymphatic clearance of Hb; and (3) enhanced lymphatic drainage of Hb was accompanied by upregulation of VEGFR‐3 on the brain surface, but not lymphatic vessel proliferation.

After SAH, extravasated blood components including free Hb activate inflammatory cells such as macrophages and microglia to induce neuroinflammation and EBI.^2^, ^3^ Extravasated blood components and neuroinflammation also upregulate a matricellular protein TNC, which further aggravates neuroinflammation and EBI through multiple mechanisms.^2^, ^3^ As these inflammatory cells possess ETR_A_,^14^ ETR_A_ antagonists are expected to suppress neuroinflammation. In fact, another endothelin receptor antagonist bosentan with a slightly higher affinity for ETR_A_ than endothelin B receptor was reported to inhibit microglial activation in mice exhibiting self‐injurious behavior.^21^ This study first demonstrated that intrathecal administration of a selective ETR_A_ antagonist Cla prevented post‐SAH activation of perivascular macrophages and microglia in mice. On the other hand, in this study, because Cla administration increased lymphatic excretion of Hb and reduced intracranial Hb, the improvement in neuroinflammation may not be attributable to the inhibition of ETR_A_ but may be a secondary effect of the reduction in Hb.

The mechanism by which lymphatic drainage of Hb increased in this study is unclear. In SAH, glymphatic and meningeal lymphatic functions are impaired, preventing effective lymphatic drainage of Hb, which may worsen EBI.^22^, ^23^ Recently, new findings have been discovered regarding the extracranial CSF drainage mechanism, such as brain lymphatic vessels that were previously thought to be non‐existent, being expected to help elucidate the pathology of various diseases.^22^, ^23^, ^24^, ^25^ In addition, TNC, one of the causative agents of EBI,^2^, ^4^ was reported to inhibit lymphangiogenesis in extracranial organs,^26^ although there is no direct evidence of TNC‐mediated regulation of intracranial lymphatics in a SAH model. As maintaining or improving lymphatic drainage is thought to be important for reducing post‐SAH Hb toxicity, we first hypothesized that Cla administration would suppress TNC expression, thereby increasing intracranial lymphatic vessels and promoting extracranial drainage of Hb. However, immunohistochemistry 48 hours after SAH failed to reveal increased lymphatic vessels by Cla treatment, although TNC was downregulated and VEGFR‐3 was upregulated. The most potent and specific prolymphangiogenic pathway is VEGFR‐3−dependent signaling activated by VEGF‐C, which is produced by macrophages and dendritic cells during neuroinflammation.^25^, ^27^ TNC was reported to be a spatiotemporal negative regulator of lymphangiogenesis and to directly inhibit lymphangiogenesis induced by VEGF‐C through activating p38 mitogen−activated protein kinase via binding to integrin αvβ1 on lymphatic endothelial cells in mouse skin.^26^ Although the relationship between TNC and the VEGF‐C receptor VEGFR‐3 remains unknown, it is possible that TNC may also inhibit post‐SAH upregulation of VEGFR‐3 via interaction between receptors and without affecting VEGF‐C expression. As a result, as shown in this study, downregulation of TNC may have increased VEGFR‐3 expression without altering VEGF‐C expression. Another possibility is that selective inhibition of ETR_A_ by Cla may lead to relative activation of endothelin B receptors and a subsequent increase in both VEGF‐C and VEGFR‐3, causing increased lymphatic vessels.^28^ However, increased VEGF‐C expression was not shown in this study. Recently, the importance of the VEGFR‐3 signaling in lymphatic physiology has been emphasized independently of VEGF‐C,^29^ but this study did not show any association between VEGFR‐3 expression and lymphangiogenesis.

Despite VEGFR‐3 upregulation, intradural lymphangiogenesis was not observed in this study. There are still limited reports on intracranial lymphatic vessel proliferation in animal models of stroke. Reportedly, meningeal lymphangiogenesis occurred from days 10 to 14 after intracerebral hemorrhage in mice.^6^ Interventional treatment with simvastatin or cilostazol accelerated lymphatic vessel proliferation in the subarachnoid space to day 3 and promoted blood clearance after intracerebral hemorrhage in rats or mice, respectively.^6^, ^30^ In a mouse model of transient middle cerebral artery occlusion, VEGF‐C/VEGFR‐3 signaling−induced lymphangiogenesis was reported to begin mainly on the brain surface near the cribriform plate by day 3, peak at day 7, and regress by day 14.^7^, ^31^ After SAH, lymphatic drainage and the glymphatic system are often impaired, possibly attributable to obstruction by blood clots.^3^ However, it is unclear whether intracranial lymphangiogenesis occurs after SAH as a compensatory mechanism for post‐SAH CSF circulatory disorders. Chen et al.^32^ reported that lymph flow in the meningeal lymphatics increased and served as the route for the drainage of erythrocytes, but post‐SAH increased lymph flow was not associated with significant lymphangiogenesis and lymphangiectasia of the meningeal lymphatics at day 7 after SAH in mice. However, meningeal lymphatic regression by VEGFR‐3 inhibition aggravated neurological outcomes and microglial activation at day 7 after SAH in mice.^32^ Although this study was unable to demonstrate lymphatic vessel proliferation 48 hours after SAH, it is possible that the examination was conducted too early and should be reexamined at a later time point. The principal downstream effectors of VEGFR‐3 signaling are phosphoinositide 3‐kinase–dependent activation of protein kinase B (Akt) and extracellular signal‐regulated kinase 1/2,^29^ and the former has been suggested to protect the structure and function of the glymphatic system.^30^ Although this study did not show an increase in lymphatic vessels, it is possible that Cla treatment enhanced VEGFR‐3 signaling, thereby alleviating glymphatic system dysfunction and contributing to the promotion of lymphatic drainage of Hb in an acute phase of SAH in mice.

It is well known that the function of meningeal lymphatic drainage can be affected by the glymphatic system function.^33^, ^34^ It has been reported that brain border macrophages (perivascular and leptomeningeal macrophages) play important roles in CSF dynamics through perivascular spaces in the glymphatic system and CSF drainage to meningeal lymphatic vessels via the regulation of extracellular matrix components.^33^, ^34^ However, under SAH, as perivascular macrophages caused perivascular inflammation, restricted arteriolar pulsation and compromised CSF flow, pharmacological depletion of perivascular macrophages was effective in treating CSF circulatory disturbance and accumulation at 48 hours after SAH in mice.^15^ In this study, Cla treatment also reduced perivascular macrophages, which may have led to improvement or enhancement in lymphatic drainage of Hb.

Glymphatic function after SAH may also be improved by increasing arterial pulsatility, which is the key force driving glymphatic flow.^35^ Intravenous infusion of Cla can significantly prevent cerebral arterial to arteriolar constriction and improve their pulsatility after SAH in a clinical setting.^8^, ^36^ According to several experimental studies using SAH models in mice or rats, on the other hand, intravenous administration of Cla (10–178 mg/kg BW) failed to exert neuroprotective effects because Cla did not sufficiently cross the blood–brain barrier.^37^, ^38^, ^39^, ^40^, ^41^ Therefore, it is expected that intrathecal administration of Cla may more effectively inhibit the activation of pericyte ETR_A_ after SAH than the intravenous infusion,^42^ which may result in more increased pulsatility of cerebral arteries and arterioles, leading to the enhanced lymphatic drainage of Hb observed in this study. The reduction in perivascular macrophages due to intrathecal Cla treatment may also have increased arteriolar pulsation, which may have contributed to the promotion of lymphatic drainage of Hb.^15^ However, in a study using the prechiasmatic cisternal injection model of SAH in rats, intrathecal administration of Cla (0.38 μg/kg BW/dose, administered intermittently six times between 6 and 60 hours after the model creation) did not show neuroprotection,^43^ although the administered dose was much smaller than this study (8–40 μg/kg BW, administered continuously from 30 minutes after modeling). To demonstrate that intrathecal administration of Cla is more effective than intravenous administration in removing Hb from the CSF and in providing neuroprotection, animal studies directly comparing these two administration methods are needed.

This study has limitations, including the use of only male animals and the fact that the mechanism of lymphatic drainage promotion after intrathecal administration of Cla has not been fully elucidated. The use of male‐biased research animals is distinct from clinical situations.^44^, ^45^ This exploratory study was performed only in male mice as an initial exploration that now requires future validation across sexes. However, this study is valuable in that it is the first to demonstrate that if a CSF concentration that is 10% of the blood concentration obtained when Cla is administered clinically can be achieved, lymphatic excretion of Hb can be promoted from the acute phase after SAH, neuroinflammation can be suppressed, and it is effective in treating EBI. The authors hope that this research will lead to the development of new treatments for EBI after SAH.

## Sources of Funding

This work was funded by the Japan Society for the Promotion of Science (grant number JP23K15644) to Dr. Kawakita and was partially supported by a research grant from Nxera Pharma to Dr. Suzuki. The authors declare that no commercial entity was involved in the design, conduct, analysis, interpretation, or publication of this research.

## Disclosures

Dr. Suzuki serves as a consultant to Nxera Pharma. The other authors declare that they have no conflict of interest.

## Supporting information

Data S1

Table S1
Figures S1‐S2

Unedited Gels
ARRIVE checklist
