# Supplementary material for: Neuroprotection and Enhanced Hemoglobin Excretion by Intrathecal Clazosentan in Mice With Subarachnoid Hemorrhage
Source: J Am Heart Assoc. 2026 Aug 7;15(16):e049695. doi: 10.1161/JAHA.126.049695 (PMC13695748; doi:10.1161/JAHA.126.049695)
Supplement: Supplementary file 1 [file JAH3-15-e049695-s001.pdf]

UNEDITED GELS

Fig. 2

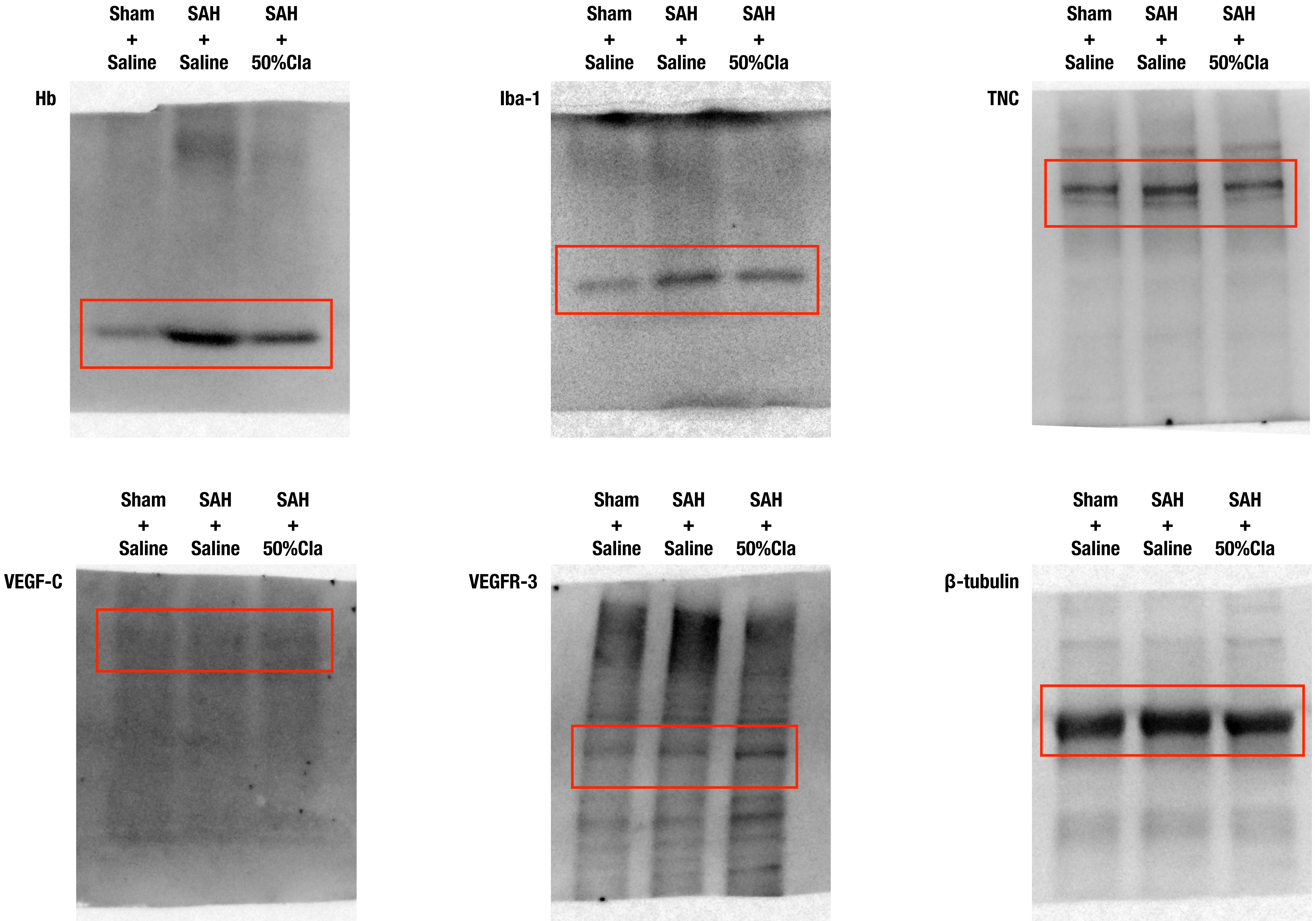

Sham + Saline
SAH + Saline
SAH + 50%Cla
Hb
Iba-1
TNC
VEGF-C
VEGFR-3
β-tubulin

Fig. 3

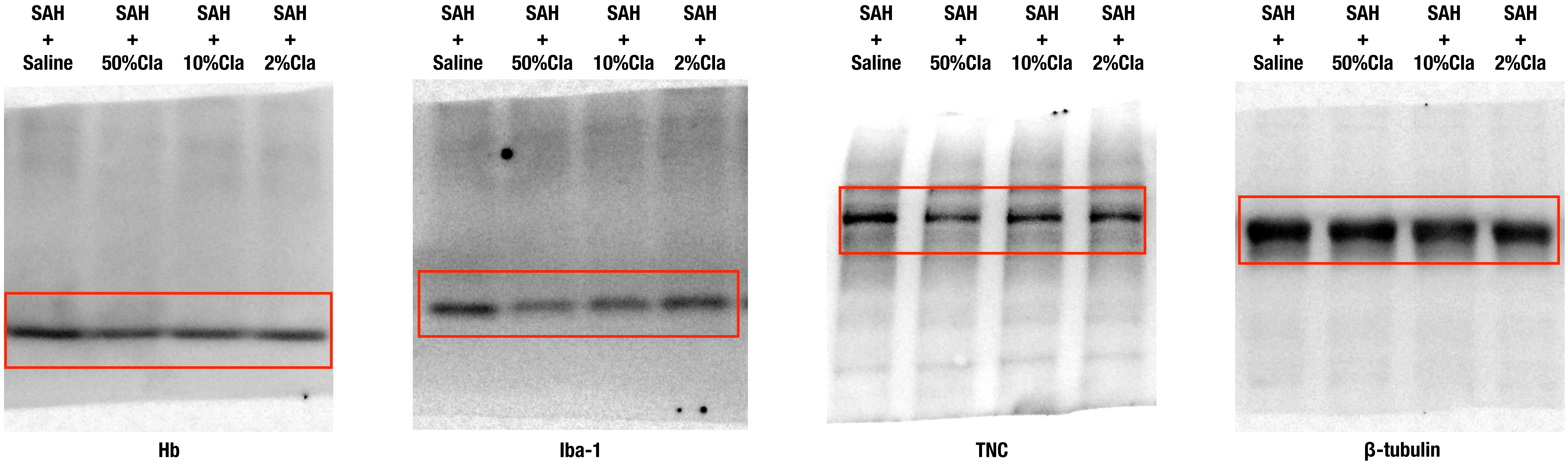
SAH + Saline
SAH + 50%Cla
SAH + 10%Cla
SAH + 2%Cla
SAH + Saline
SAH + 50%Cla
SAH + 10%Cla
SAH + 2%Cla
SAH + Saline
SAH + 50%Cla
SAH + 10%Cla
SAH + 2%Cla
SAH + Saline
SAH + 50%Cla
SAH + 10%Cla
SAH + 2%Cla
Hb
Iba-1
TNC
β-tubulin
