# Supplementary material for: Neuroprotection and Enhanced Hemoglobin Excretion by Intrathecal Clazosentan in Mice With Subarachnoid Hemorrhage
Source: J Am Heart Assoc. 2026 Aug 7;15(16):e049695. doi: 10.1161/JAHA.126.049695 (PMC13695748; doi:10.1161/JAHA.126.049695)
Supplement: Supplementary file 2 [file JAH3-15-e049695-s002.pdf]

SUPPLEMENTAL MATERIAL

Data S1. Major Resources Table

In order to allow validation and replication of experiments, all essential research materials listed in the Methods should be included in the Major Resources Table below. Authors are encouraged to use public repositories for protocols, data, code, and other materials and provide persistent identifiers and/or links to repositories when available. Authors may add or delete rows as needed.

Mouse Models Used in This Study

Strain	Vendor or Source	Background Strain	Sex	Other Information (e.g. breeding scheme for F1, F2…if applicable)	Persistent ID / URL
	SLC, Hamamatsu, Japan	C57BL/6NCrSlc	M	10–12 weeks old, weighting 25–30g	https://www.jslc.co.jp/pdf/mouse/2020/007_C57BL_6NCrSlc.pdf

Antibodies

Target antigen	Vendor or Source	Catalog #	Working concentration	Lot # (preferred but not required)	Persistent ID / URL
Mouse Hemoglobin	LSBio, Seattle, WA	LS-B12169	1:2000 (Western blotting); 1:100 (immunostaining)		https://www.lsbio.com/antibodies/ihc-plus-hemoglobin-antibody-elisa-ihc-wb-western-ls-b12169/359647
Iba-1	Abcam, Cambridge, MA	ab178847	1:1000 (Western blotting); 1:2000 (immunostaining)		https://www.abcam.co.jp/products/primary-antibodies/iba1-antibody-epr16589-microglia-marker-ab178847
Tenascin-C			1:1000 (Western blotting)		El-Karef A, et al. Deficiency of tenascin-C attenuates liver fibrosis in immune-mediated chronic hepatitis in mice. J Pathol. 2007;211:86–94
VEGF-C	Abcam, Cambridge, MA	ab9546	1:500 (Western blotting)		https://www.abcam.co.jp/products/primary-antibodies/vegfc-antibody-ab9546
VEGFR-3	Abcam, Cambridge, MA	ab27278	1:1000 (Western blotting); 1:100 (immunostaining)		https://www.abcam.co.jp/products/primary-antibodies/vegf-receptor-3-antibody-ab27278
β-Tubulin	Cell Signaling Technology, Danvers, MA	2146	1:4000		https://www.cellsignal.com/products/primary-antibodies/beta-tubulin-antibody/2146
LYVE-1	Abcam, Cambridge, MA	ab14917	1:100 (immunostaining)		https://www.abcam.co.jp/products/primary-antibodies/lyve1-antibody-bsa-and-azide-free-ab14917
Rabbit polyclonal immunoglobulin G	Vector Laboratories, Newark, CA		1:200 (immunostaining)		https://vectorlabs.com/products/rtu-biotinylated-goat-anti-rabbit-igg/

Other

Description	Source / Repository	Persistent ID / URL
Clazosentan	Nxera Pharma, Tokyo, Japan	
ALZET minipump	Durect Corp, Cupertino, CA	Model #2001D

Table S1. The number of animals and mortality in each experiment

Experiment 1

Modeling	Pre-randomization − 30 minutes	Treatment	Post-randomization − 24 hours
Sham (n=12)	0 / 12 (0%)	Saline (n=12)	0 / 12 (0%) 12 sacrifices
	Total mortality = 0 / 12 (0%)		
SAH (n=29)	2 / 29 (6.9%)	Saline (n=15)	3 / 15 (20.0%) 12 sacrifices
		50% Cla (n=12)	0 / 12 (0%) 12 sacrifices
	Total mortality = 5 / 29 (17.2%)		

Experiment 2

Modeling	Pre-randomization − 30 minutes	Treatment	Post-randomization − 24 hours
SAH (n=32)	4 / 32 (12.5%)	Saline (n=8)	2 / 8 (25.0%) 6 sacrifices
		50% Cla (n=6)	0 / 6 (0%) 6 sacrifices
		10% Cla (n=6)	0 / 6 (0%) 6 sacrifices
		2% Cla (n=8)	2 / 8 (25.0%) 6 sacrifices
	Total mortality = 8 / 32 (25.0%)		

Experiment 3

Modeling	Pre-randomization − 30 minutes	Treatment	Post-randomization		
			− 24 hours	− 48 hours	Total
Sham (n=6)	0 / 6 (0%)	Saline (n=6)	0 / 6 (0%) 0 sacrifice	0 / 6 (0%) 6 sacrifices	0 / 6 (0%)
	Total mortality = 0 / 6 (0%)				
SAH (n=30)	3 / 30 (10.0%)	Saline (n=11)	1 / 11 (9.1%) 0 sacrifice	3 / 10 (30.0%) 7 sacrifices	4 / 11 (36.4%)
		10% Cla (n=7)	1 / 7 (14.3%) 0 sacrifice	0 / 6 (0%) 6 sacrifices	1 / 7 (14.3%)
		2% Cla (n=9)	2 / 9 (22.2%) 0 sacrifice	1 / 7 (14.3%) 6 sacrifices	3 / 9 (33.3%)
	Total mortality = 11 / 30 (36.7%)				

Data are presented as the number of mice that died / total number of mice (mortality, %). “Sacrifice” indicates the number of mice sacrificed. No significant differences in mortality were observed between SAH groups (chi-square test or Fisher’s exact test). Cla, clazosentan.

Figure S1. Experimental designs.

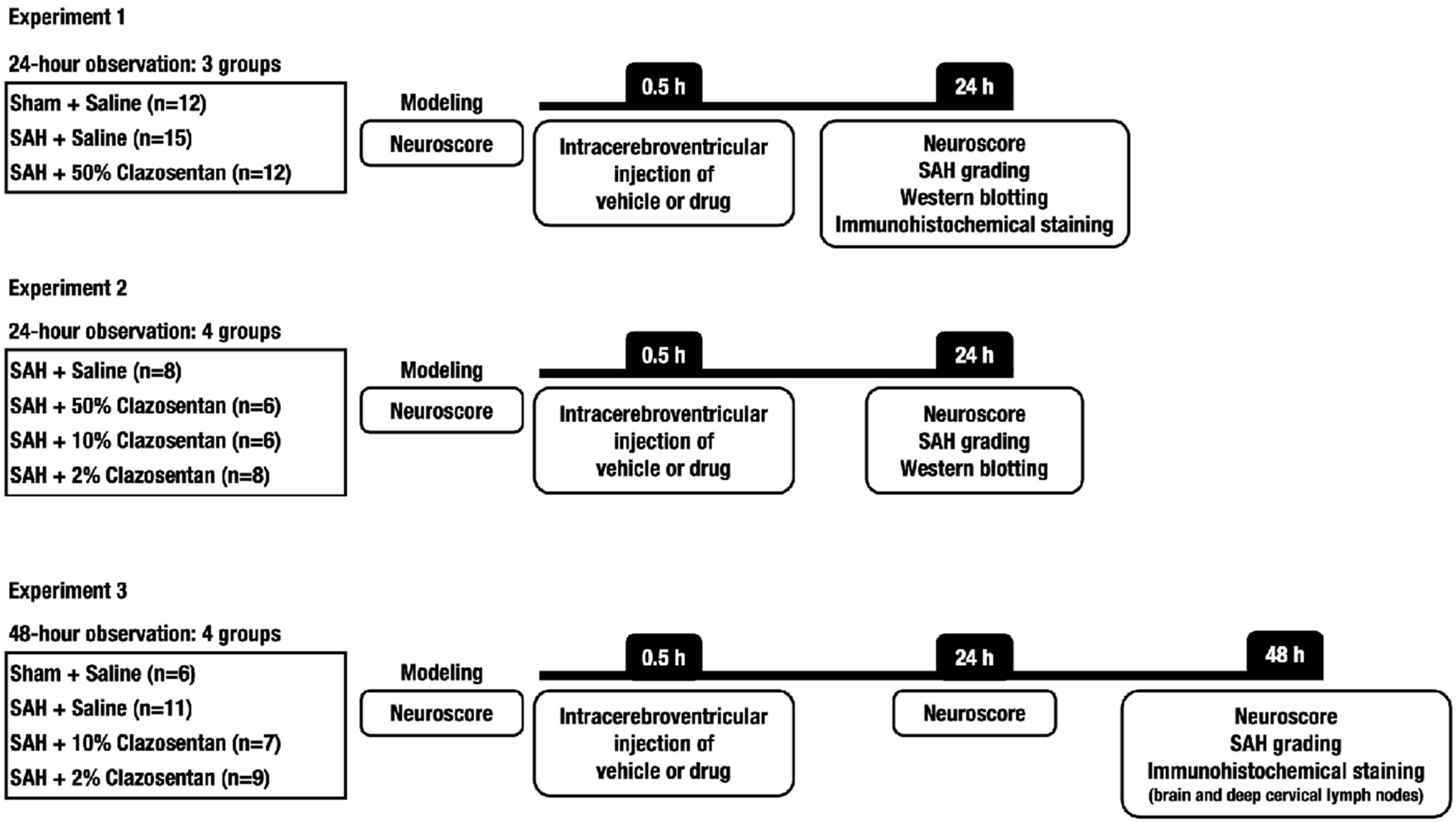

Experiment 1 is designed to evaluate the neuroprotective effects of clazosentan after subarachnoid hemorrhage (SAH). Experiment 2 is designed to examine the dose-response relationship of clazosentan to SAH toxicity. Experiment 3 is designed to confirm the neuroprotective effect of clazosentan and to examine its relationship to lymphatic drainage.

Figure S2. Four contiguous regions of the right (blood-injection side) temporal cortex on brain slices cut coronally 1.0mm posterior to bregma, which were evaluated for immunohistochemical staining in this study.

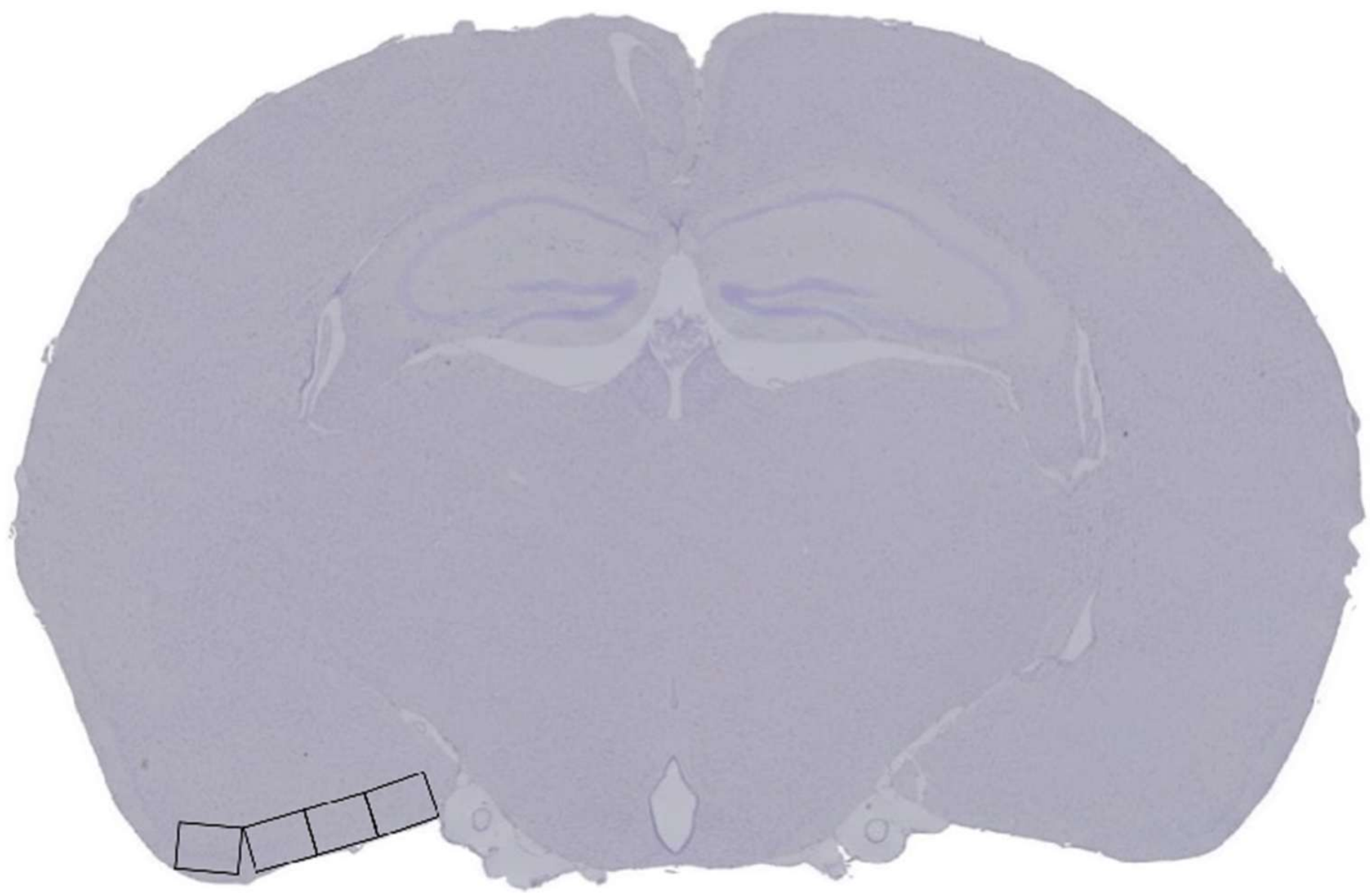

ARRIVE GUIDELINES

The ARRIVE guidelines (https://arriveguidelines.org/) are a checklist of recommendations to improve the reporting of research involving animals. Key elements of the study design should be included below to better enable readers to scrutinize the research adequately, evaluate its methodological rigor, and reproduce the methods or findings.

Study Design

Groups	Sex	Age	Number (prior to experiment)	Number (after termination)	Littermates (Yes/No)	Other description
Sham (Control)	M	10−12 weeks old	18	18	No	
SAH Saline	M	10−12 weeks old	34	25	No	
SAH 50%Cla	M	10−12 weeks old	18	18	No	
SAH 10%Cla	M	10−12 weeks old	13	12	No	
SAH 2%Cla	M	10−12 weeks old	17	12	No	

Sample Size: Please explain how the sample size was decided Please provide details of any a *prior* sample size calculation, if done.

Lines 125 to 127: The sample size of this study (n=6 or 12 per group) was determined based on a large effect size (≥ 0.80) expected from preliminary data and previous studies using similar models.[5,15]

Inclusion Criteria
Line 170: All animals were used, and no exclusions were made.

Exclusion Criteria
Line 170: All animals were used, and no exclusions were made.

Randomization
Lines 127 to 128: After modeling, animals were immediately randomized by lottery until the final group size reached the planned number of experiments.

Blinding
Lines 128 to 129: Data collection and analysis were performed in a blinded manner.
