# Supplementary material for: Neuroprotection and Enhanced Hemoglobin Excretion by Intrathecal Clazosentan in Mice With Subarachnoid Hemorrhage
Source: J Am Heart Assoc. 2026 Aug 7;15(16):e049695. doi: 10.1161/JAHA.126.049695 (PMC13695748; doi:10.1161/JAHA.126.049695)
Supplement: Supplementary file 3 [file JAH3-15-e049695-s003.pdf]

The ARRIVE reporting checklist

For checking that articles describing *in vivo* animal experiments can be understood and used by everyone

How to use this reporting checklist

This reporting checklist allows authors to demonstrate that their manuscripts adhere to the ARRIVE reporting guideline.

If you have not used a reporting guideline before, read about how and why to use them and check whether ARRIVE is the most applicable reporting guideline for your work.

Reporting guidelines are most useful when used early in research. When writing a manuscript or application, consider using the full guidance where you'll find explanations and examples for each item.

After writing, demonstrate adherence by completing this checklist:

1. Specify where each item is described (see Note 1).
2. Cite this checklist (See Note 2).
3. Include your completed checklist as a supplement when submitting to a journal so that future readers can use it to find information.

	Item Description	Location (or reason for not reporting)
**Essential 10**		
1. Study Design		
1a. The groups being compared	For each experiment, describe the groups being compared, including control groups. If you did not use a control group, explain why.	Lines 175–192
1b. The experimental unit	Describe the experimental unit (e.g., a single animal, litter, or cage of animals).	Lines 125–128
2. Sample Size		
2a. Number of Experimental Units	Specify the exact number of experimental units allocated to each group, and the total number in each experiment. Also indicate the total number of animals used.	Table S1
2b. Sample Size Justification	Explain how the sample size was decided. Provide details of any a priori sample size calculation, if done.	Lines 130–132
3. Inclusion and Exclusion Criteria		
3a. Inclusion and	Describe any criteria used for including or excluding animals (or experimental units) during the	Line 175

exclusion criteria	experiment, and data points during the analysis. Specify if these criteria were established a priori. If no criteria were set, state this explicitly.	
3b. Exclusions and Attritions	For each experimental group, report any animals, experimental units, or data points not included in the analysis and explain why. If there were no exclusions, state so.	Table S1
3c. Numbers analysed	For each analysis, report the exact value of *n* in each experimental group.	Figures
4. Randomisation		
4a. Randomisation Use	State whether randomisation was used to allocate experimental units to control and treatment groups. If done, provide the method used to generate the randomisation sequence.	Lines 132–133
4b. Confounders	Describe the strategy used to minimise potential confounders such as the order of treatments and measurements, or animal/cage location. If confounders were not controlled, state this explicitly.	Lines 132–134
5. Blinding/Masking	Describe who was aware of the group allocation at the different stages of the experiment (during the allocation, the conduct of the experiment, the outcome assessment, and the data analysis).	Lines 133–134
6. Outcome Measures		
6a. Outcome Measures	Clearly define all outcome measures assessed (e.g., cell death, molecular markers, or behavioural changes).	Lines 194–240
6b. Primary Outcome Measure	For hypothesis-testing studies, specify the primary outcome measure, i.e., the outcome measure that was used to determine the sample size.	Lines 130–132
7. Statistical Methods		
7a. Statistical Methods used for each Analysis	Provide details of the statistical methods used for each analysis, including software used.	Lines 242–253
7b. Statistical Assumptions	Describe any methods used to assess whether the data met the assumptions of the statistical approach, and what was done if the assumptions were not met.	Lines 244–250
8. Experimental Animals		
8a. Species-appropriate Details	Provide species-appropriate details of the animals used, including species, strain and substrain, sex, age or developmental stage, and, if relevant, weight.	Lines 125–126
8b. Further	Provide further relevant information on the	N/A

Information	provenance of animals, health/immune status, genetic modification status, genotype, and any previous procedures.	
9. Experimental Procedures		
9a. What was done	What was done, how it was done, and what was used.	Lines 136–240
9b. When and how often procedures were conducted	For each experimental group, including controls, describe **when and how often** procedures were performed.	Lines 175–192
9c. Where procedures were conducted	For each experimental group, including controls, describe **where** procedures were conducted (including detail of any acclimatisation periods).	Lines 125–128
9d. Why procedures were done	For each experimental group, including controls, describe **why** procedures were conducted.	Lines 175–192
10. Results		
10a. Summary/Descriptive Statistics per group	For each experiment conducted, including independent replications, report a summary/descriptive statistics for each experimental group, with a measure of variability where applicable (e.g., mean and SD, or median and range).	Lines 324–330
10b. Effect sizes and confidence intervals	For each experiment conducted, including independent replications, report the effect size with a confidence interval, if applicable.	N/A
**Recommended Set**		
11. Abstract	Provide an accurate summary of the research objectives, animal species, strain and sex, key methods, principal findings, and study conclusions.	Lines 24–51
12. Background		
12a. Rationale	Include sufficient scientific background to understand the rationale and context for the study, and explain the experimental approach.	Lines 83–115
12b. Species and model	Explain how the animal species and model used address the scientific objectives and, where appropriate, the relevance to human biology.	Lines 83–115
13. Objectives	Clearly describe the research question, research objectives and, where appropriate, specific hypotheses being tested.	Lines 111–115
14. Ethical statement	Provide the name of the ethical review committee or equivalent that has approved the use of animals in this study and any relevant licence or protocol numbers (if applicable). If ethical approval was not sought or granted, provide a justification.	Lines 123–125
15. Housing and husbandry	Provide details of housing and husbandry conditions, including any environmental enrichment.	Lines 125–128

16. Animal Care and Monitoring		
16a. Reducing pain, suffering, and distress	Describe any interventions or steps taken in the experimental protocols to reduce pain, suffering, and distress.	Lines 152–155
16b. Adverse events	Report any expected or unexpected adverse events.	Table S1
16c. Humane endpoints	Describe the humane endpoints established for the study, the signs that were monitored, and the frequency of monitoring. If the study did not set humane endpoints, state this.	Lines 173–174, and 194–196
17. Interpretation/ scientific implications		
17a. Interpretation/scientific implications	Interpret the results, taking into account the study objectives and hypotheses, current theory, and other relevant studies in the literature.	Lines 323–439
17b. Limitations	Comment on the study limitations, including potential sources of bias, limitations of the animal model, and imprecision associated with the results.	Lines 430–434
18. Generalisability/translation	Comment on whether, and how, the findings of this study are likely to generalise to other species or experimental conditions, including any relevance to human biology (where appropriate).	Lines 432–439
19. Protocol registration	Provide a statement indicating whether a protocol (including the research question, key design features, and analysis plan) was prepared before the study, and if and where this protocol was registered.	N/A
20. Data Access	Provide a statement describing if and where study data are available.	Lines 118–120
21. Declaration of interests		
21a. Conflicts of interests	Declare any potential conflicts of interest, including financial and nonfinancial. If none exist, this should be stated.	Lines 457–458
21b. Funding	List all funding sources (including grant identifier) and the role of the funder(s) in the design, analysis, and reporting of the study.	Lines 451–454

1 How to specify where content is

Tell the reader where they can find information. E.g.,

- Results; paragraph 2
- Methods, Participants; paragraphs 1 & 2.

- Table 3
- Supplement B, para. 4

If you have chosen not to describe an item, explain why. You can do this in the checklist, or as a note below it.

You can describe items in the article body, or in tables, figures, or supplementary materials, and should prioritize items you feel are most important to your intended audience. The order of items in your manuscript does not need to match the order of items in this checklist. You can decide how best to structure your work.

2 How to cite

Describe how you used ARRIVE at the end of your Methods section, referencing the resources you used e.g.,

> 'We used the ARRIVE reporting guideline(1) to draft this manuscript, and the ARRIVE reporting checklist(2) when editing, included in supplement A'

If you use a reporting checklist, remember to include it as a supplement when publishing so that readers can easily find information and see how you have interpreted the guidance.

1. Sert NP, Hurst V, Ahluwalia A, Alam S, Avey MT, Baker M, et al. The ARRIVE guidelines 2.0: Updated guidelines for reporting animal research. PLOS Biology [Internet]. 2020 Jul;18(7):e3000410. Available from: https://journals.plos.org/plosbiology/article?id=10.1371/journal.pbio.3000410

2. Sert NP, Hurst V, Ahluwalia A, Alam S, Avey MT, Baker M, et al. The ARRIVE reporting checklist. In: Harwood J, Albury C, Beyer J de, Schlüssel M, Collins G, editors. The EQUATOR network reporting guideline platform [Internet]. The UK EQUATOR Centre; 2025. Available from: https:/resources.equator-network.org/reporting-guidelines/arrive/arrive-checklist.docx
